# Comparative associations between anticholinergic burden and emergency department visits for anticholinergic adverse events in older Korean adults: a nested case-control study using national claims data for validation of a novel country-specific scale

**DOI:** 10.1186/s40360-020-00467-6

**Published:** 2021-01-07

**Authors:** Sunghee Hwang, Jee Eun Chung, Kwanghee Jun, Young-Mi Ah, Kwang-Il Kim, Ju-Yeun Lee

**Affiliations:** 1 College of Pharmacy and Institute of Pharmaceutical Science and Technology, Hanyang University, Ansan, Gyeonggi-do Republic of Korea; 2 College of Pharmacy and Research Institute of Pharmaceutical Sciences, Seoul National University, Seoul, Republic of Korea; 3 College of Pharmacy, Yeungnam University, Gyeongsan-si, Gyeongsangbuk-do Republic of Korea; 4 Department of Internal Medicine, Seoul National University Bundang Hospital, Seognam-si, Gyeonggi-do Republic of Korea

**Keywords:** Anticholinergic syndromes, Cholinergic antagonists, Emergency medical services, Geriatrics

## Abstract

**Background:**

Considering the limited generalizability of previous anticholinergic burden scales, the Korean Anticholinergic Burden Scale (KABS) as a scale specific to the Korean population was developed. We aimed to validate the KABS by detecting the associations between high anticholinergic burden, measured with the KABS, and emergency department (ED) visits compared to the pre-existing validated scales in older Korean adults.

**Methods:**

A nested case-control study was conducted using national claims data. The cases included the first anticholinergic ED visits between July 1 and December 31, 2016. Anticholinergic ED visits were defined as ED visits with a primary diagnosis of constipation, delirium, dizziness, fall, fracture, or urinary retention.

Propensity score-matched controls were identified. Average daily AB scores during 30 days before the index date were measured. Multivariate logistic regression analyses were performed.

**Results:**

In total, 461,034 were included. The highest proportion of those with high AB was identified with KABS (5.0%). Compared with those who had a KABS score of 0, older adults with a score ≥ 3 were at higher risk for overall anticholinergic ED visits (aOR, 1.62, 95% CI, 1.53–1.72), as well as visits for falls/fractures (aOR: 1.54, 95% CI: 1.40–1.69), dizziness (aOR: 1.44, 95% CI: 1.30–1.59), delirium (aOR: 2.96, 95% CI: 2.28–3.83), constipation (aOR: 1.84, 95% CI: 1.68–2.02), and urinary retention (aOR: 2.13, 95% CI: 1.79–2.55). High AB by KABS showed a stronger association with overall anticholinergic ED visits and visits due to delirium and urinary retention than those by other scales.

**Conclusions:**

In conclusion, KABS is superior to pre-existing scales in identifying patients with high AB and predicting high AB-related ED visits in older Korean adults.

**Supplementary Information:**

The online version contains supplementary material available at 10.1186/s40360-020-00467-6.

## Background

Anticholinergic drugs are often prescribed for the treatment of various diseases or for the relief of various symptoms, but they are often characterized as inappropriate for older adults [1]. Anticholinergic burden is defined as the cumulative effects of taking multiple drugs with anticholinergic properties [2]. A high anticholinergic burden can cause a variety of negative consequences, including cognitive impairment, confusion, delirium, falls, dry mouth, constipation and urinary retention [2].

Anticholinergic burden has become a prominent indicator used to evaluate the quality of prescribing practices in geriatric pharmacotherapy [3]. This assessment is used to recognize the risk of drug-related complications and to reduce unnecessary anticholinergic prescriptions in medication reviews for polypharmacy in older adults. A study conducted in the United Kingdom by Tay et al. [4] found that simply measuring the AB then reporting it to the physician could reduce anticholinergic prescriptions. A study by Ailabouni et al. [5] showed that anticholinergics or sedatives could be deprescribed after a pharmacist performed a medication review.

Several assessment scales, such as the Anticholinergic Risk Scale (ARS) [6], the Anticholinergic Cognitive Burden Scale (ACB) [7], and the Anticholinergic Drug Scale (ADS) [8] have been developed to evaluate anticholinergic burden and predict potential anticholinergic adverse effects [9, 10]. However, these scales were difficult to apply to Korean population due to their limited generalizability and the inconsistencies of listed medications and their anticholinergic potency scores. The limited generalizability was related to the difference in prescribing practices as well as the availability of the medications between countries. In addition, these differences could have occurred with the change of times after development of the scale [11]. For these reasons, there is no consensus on which scoring system is most useful in clinical settings to date [12]. To overcome these limitations, we developed the Korean Anticholinergic Burden Scale (KABS) as a scale specific to the Korean population, through a modified Delphi process using the previously developed scales but with the addition of Korean-specific anticholinergic medications [13]. This scale re-evaluated the medications with mismatched anticholinergic scores in the previous scales and included the anticholinergic medications commonly used in South Korea that were not rated in existing scale [13]. Previous studies reported that identification of populations at risk for adverse anticholinergic effects and the predictive validity for negative clinical outcomes may depend on the scales applied [10, 14–16]. Therefore, before adopting the KABS in clinical practice, it is essential to confirm its practicality by evaluating the association between KABS and negative clinical outcomes.

The preponderance of previous studies focused on the chronic, central nervous system adverse effects, such as dementia or cognitive impairment, or falls as the marker of negative outcomes secondary to high anticholinergic burden. However, from the perspectives of patients and healthcare systems alike, it is important to include other severe or acute adverse effects, including urinary retention, constipation, dizziness, delirium, and a fall or fracture, leading to ED visits as they increase the economic and cost burden [17].

Based on these previous findings, we aimed to validate the novel KABS by detecting the associations between high anticholinergic burden, measured with the KABS, and ED visits related to anticholinergic adverse effects (constipation, dizziness, delirium, falls, fractures, or urinary retention) compared to the pre-existing validated scales in Korean older adults.

## Methods

### Study design and population

We conducted a nested case-control study using an older adult patient sample (APS) dataset provided by the Health Insurance Review and Assessment Service (HIRA), the HIRA-APS 2016. Under the Korean national health insurance system, which includes 98% of the Korean population, HIRA originally receives claims data to evaluate claims for medical services that healthcare providers deliver to patients prior to reimbursement. Using stratified random sampling method, HIRA provided the HIRA-APS, which contained approximately one million individuals (20%) randomly selected from all patients aged 65 years or over in 2016 [18] . This dataset exhibits 95% concordance with the actual population, demonstrating a high level of representativeness. The HIRA claims data include basic socio-demographic information, specific information on health service provided including procedures, prescription data and diagnostic information.

**Fig. 1:**
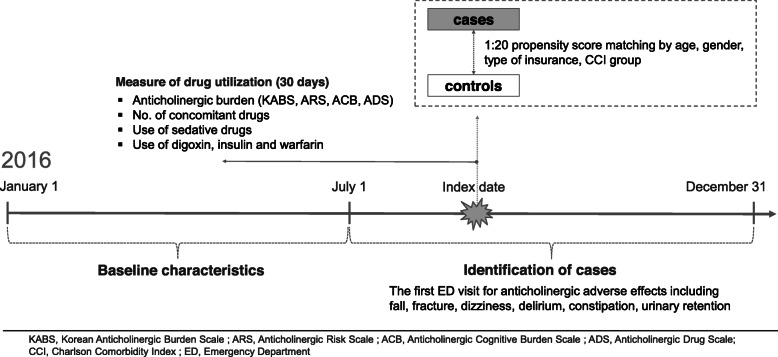
Study design

The study design is depicted in Fig. 1. The cases consisted of older adults who visited an ED due to anticholinergic adverse effects, an anticholinergic ED visits, from July 1 to December 31, 2016. Anticholinergic adverse effects were identified using primary diagnostic codes related to constipation, dizziness, delirium, falls, fractures, or urinary retention (Additional file 1) and the first anticholinergic ED visit was set as the index date. Patients who had anticholinergic ED visits prior to the index date were excluded. The controls were matched to cases using a propensity score (1:20) according to age, sex, insurance type, and Charlson Comorbidity Index (CCI) score (0, 1–2, ≥ 3) among those who did not visit an ED during 2016. Propensity score matching was performed using a greedy algorithm with a caliper width equal to 0.2 standard deviations of the logit of the propensity score without replacement. We assigned the index date of control as the date at which the event occurred for the matched cases.

This study obtained an ethical approval from the Institutional Review Board of Hanyang University (No. HYI-17-007-1). Informed consent was waived because we used encrypted claims data.

### Measurement of anticholinergic medication exposure

A patient’s anticholinergic burden was calculated as the average daily anticholinergic burden score for all prescribed drugs during the 30 days before the index date using the KABS [13], ARS [6], ACB [7] and ADS [8]. All four scales employ a 4-point scale from 0, of limited or none, to 3, of very strong, to rank the anticholinergic burden. The comparable list of anticholinergic drugs included in each of the four scales is presented in Additional file 2. Topical agents were excluded. We converted the dose of each medication to the defined daily dose (DDD) assigned by the WHO [19]. The average daily anticholinergic burden score was calculated based on the pre-rated anticholinergic potency, dosage and treatment duration as described in the previous study [20]. For this analysis, average daily scores were rounded to the nearest integer, and score of 3 or greater was defined as a high anticholinergic burden.

### Covariates

Baseline characteristics were collected from January to June 2016. Sociodemographic variables, including age, sex, and type of insurance were collected. Comorbid medical conditions, CCI scores, and concomitant chronic medications prescribed for more than 18 days during a month were identified as covariates. Among the concomitant chronic medications, we identified the number of medications, the use of sedatives, insulins, warfarin, and digoxin. The diagnostic codes used to identify comorbidities are presented in Additional file 1. To adjust for the increased risk of adverse effects, such as falls or fractures, due to the use of sedatives not on the list of anticholinergic burden scale [21], exposure to drugs listed on the sedative load model [22] was also evaluated. All these covariates were adjusted for in the multivariate logistic regression.

### Statistical analysis

Descriptive statistics were used to report the baseline characteristics and medication exposures. To compare the variables between the cases and control groups, we used t-test and chi-square statistics for numerical data and categorical data, respectively. A multivariate logistic regression analysis was performed to investigate the associations between anticholinergic burden and ED visits for all causes as well as for each specific cause, and the adjusted odds ratio (aOR) and 95% confidence interval (CI) were presented. Statistical significance was defined as *p* < 0.05. All statistical analyses were carried out with SAS version 9.4 (SAS Institute Inc., Cary, NC, USA).

## Results

Among a total of 1,327,455 older adults in the HIRA-APS 2016 dataset, 461,034 were included in this study. Table 1 presents the baseline characteristics and drug utilization data for the study population. The population had a mean age of 76.1 ± 7.1 years, and 39.1% were male. The mean CCI score was 2.11 ± 1.93, and diabetes mellitus (29.7%) was the most common comorbidity. There were 202,939 older adults (44.1%) taking 5 or more drugs. Forty-nine percent of the population was exposed to sedatives and the use of the high-risk medications, digoxin, insulins and warfarin were 1.4, 1.3, and 0.9%, respectively. Although the frequency of patients within groups divided by CCI score were similar between case and control, the mean CCI score was higher in case than control (2.25 ± 2.16 vs. 2.10 ± 1.92, *p* < 0.001). Pre-defined co-morbid conditions, polypharmacy, excessive polypharmacy, use of sedative drugs, digoxin, insulin and warfarin were significantly more prevalent among cases than controls (Table 1). These variables were included as covariates for adjustment in the multivariate logistic regression.

**Table 1:** Baseline characteristics of the study population (*N* = 461,034)

Characteristics	Total (*N =* 461,034)		Case (*N* = 21,954)		Control (*N* = 439,080)		*P value*
	N	(%)	N	(%)	N	(%)	
**Age, mean [SD]**	76.1	[7.1]	76.2	[7.1]	76.1	[7.1]	0.8844
65–74	204,246	(44.3)	9726	(44.3)	194,520	(44.3)	0.9743
75–84	194,220	(42.1)	9238	(42.1)	184,982	(42.1)	0.9743
≥ 85	62,568	(13.6)	2990	(13.6)	59,578	(13.6)	0.9743
**Gender, male**	180,351	(39.1)	8627	(39.3)	171,724	(39.1)	0.5819
**CCI score, mean [SD]**	2.11	[1.93]	2.25	[2.16]	2.10	[1.92]	<.0001
0	97,658	(21.2)	4658	(21.2)	93,000	(21.2)	0.9917
1–2	198,912	(43.1)	9468	(43.1)	189,444	(43.2)	0.9917
≥ 3	164,464	(35.7)	7828	(35.7)	156,636	(35.7)	0.9917
**Health insurance type**							
Health insurance	403,869	(87.6)	19,228	(87.6)	384,641	(87.6)	0.9355
Medical aid	57,165	(12.4)	2726	(12.4)	54,439	(12.4)	0.9355
**Co-morbid condition**							
Diabetes Mellitus	136,783	(29.7)	6702	(30.5)	130,081	(29.6)	0.0043
COPD	73,613	(16.0)	4126	(18.8)	69,487	(15.8)	<.0001
Cerebrovascular disease	64,855	(14.1)	3732	(17.0)	61,123	(13.9)	<.0001
Coronary artery disease	59,108	(12.8)	3319	(15.1)	55,789	(12.7)	<.0001
Liver failure	37,934	(8.2)	2215	(10.1)	35,719	(8.1)	<.0001
Congestive heart failure	30,984	(6.7)	1813	(8.3)	29,171	(6.6)	<.0001
Cancer	28,152	(6.1)	1640	(7.5)	26,512	(6.0)	<.0001
Renal failure	10,927	(2.4)	778	(3.5)	10,149	(2.3)	<.0001
Parkinson's disease	10,347	(2.2)	749	(3.4)	9598	(2.2)	<.0001
**Medication Use**							
**No. of concomitant drugs, mean [SD]**	4.4	[3.9]	5.2	[4.4]	4.4	[3.9]	<.0001
< 5	258,095	(56.0)	10,829	(49.3)	247,266	(56.3)	<.0001
Polypharmacy (5–9)	153,316	(33.3)	7580	(34.5)	145,736	(33.2)	<.0001
Excessive polypharmacy (≥ 10)	49,623	(10.8)	3545	(16.2)	46,078	(10.5)	<.0001
**Use of sedative drugs**	225,843	(49.0)	13,797	(62.9)	212,046	(48.3)	<.0001
**Use of digoxin**	6610	(1.4)	406	(1.9)	6204	(1.4)	<.0001
**Use of insulin**	6077	(1.3)	517	(2.4)	5560	(1.3)	<.0001
**Use of warfarin**	4278	(0.9)	300	(1.4)	3978	(0.9)	<.0001

*SD* standard deviation, *CCI* Charlson Comorbidity Index, *COPD* Chronic obstructive pulmonary disease

The average daily anticholinergic burden scores determined by the four different scales are delineated in Table 2. The highest exposure was identified using the ACB (58.0%), and the lowest exposure was identified using the ARS (37.6%). The proportion of patients who had a high anticholinergic burden (score ≥ 3) ascertained by the KABS, ACB, ADS, and ARS was 5.0, 4.8, 3.8, and 0.6%, respectively.

**Table 2:** Average daily anticholinergic burden score identified according to each four scales in the study population (*N =* 461,034)

Scale	Any exposure		Score 0		Score 1		Score 2		Score ≥ 3	
	N	(%)	N	(%)	N	(%)	N	(%)	N	(%)
**KABS**	249,523	(54.1)	324,457	(70.4)	86,278	(18.7)	27,436	(6.0)	22,863	(5.0)
**ARS**	173,533	(37.6)	394,441	(85.6)	54,499	(11.8)	9143	(2.0)	2951	(0.6)
**ACB**	267,386	(58.0)	295,633	(64.1)	112,597	(24.4)	30,704	(6.7)	22,100	(4.8)
**ADS**	252,078	(54.7)	322,466	(69.9)	90,167	(19.6)	30,784	(6.7)	17,617	(3.8)

*AB* anticholinergic burden, *KABS* Korean Anticholinergic Burden Scale, *ARS* Anticholinergic Risk Scale, *ACB* Anticholinergic Cognitive Burden scale, *ADS* Anticholinergic Drug Scale

As shown in Table 3, after adjusting for covariates, all 4 scales used for assessing anticholinergic burden showed clear dose-response relationships, with increased odds for ED visits due to anticholinergic adverse effects. Anticholinergic burden measured with the KABS and ADS showed dose-dependent increases in the odds of visiting the ED due to each individual anticholinergic adverse effect. Associations with all individual anticholinergic ED visits, except dizziness, were lowest when measured with the ACB. Older adults with a KABS score ≥ 3 were at higher risk for overall anticholinergic ED visits (aOR, 1.65; 95% CI, 1.56–1.75) as well as ED visit for falls or fractures (aOR: 1.53, 95% CI: 1.39–1.69), dizziness (aOR: 1.49, 95% CI: 1.34–2.65), delirium (aOR: 2.85, 95% CI: 2.20–3.69), constipation (aOR: 1.85, 95% CI: 1.69–2.03), and urinary retention (aOR: 2.16, 95% CI: 1.81–2.58) than those with a KABS score of 0. High anticholinergic burden measured with the KABS showed the strongest association with delirium and urinary retention, as well as overall anticholinergic ED visits, among those measured with the four scales.

**Table 3:** Association between anticholinergic burden measured by each scales and ED visits for anticholinergic adverse effects in older Korean adults (*N =* 461,034)

Scale and Score	Anticholinergic ED visits (***N =*** 21,954)		Fall or fracture (***N*** = 8124)		Dizziness (***N*** = 7026)		Delirium (***N*** = 719)		Constipation (***N*** = 7141)		Urinary retention (***N*** = 1878)	
	aOR^**a**^	(95% CI)	aOR^**a**^	(95% CI)	aOR^**a**^	(95% CI)	aOR^**a**^	(95% CI)	aOR^**a**^	(95% CI)	aOR^**a**^	(95% CI)
**KABS**												
0 (reference)	1.00		1.00		1.00		1.00		1.00		1.00	
1	1.30	(1.26–1.35)	1.24	(1.17–1.31)	1.23	(1.15–1.30)	1.36	(1.12–1.66)	1.38	(1.30–1.47)	1.50	(1.34–1.69)
2	1.46	(1.38–1.54)	1.37	(1.25–1.49)	1.29	(1.17–1.41)	1.91	(1.46–2.50)	1.68	(1.54–1.84)	1.68	(1.41–2.00)
≥ 3	1.62	(1.53–1.72)	1.54	(1.40–1.69)	1.44	(1.30–1.59)	2.96	(2.28–3.83)	1.84	(1.68–2.02)	2.13	(1.79–2.55)
**ARS**												
0 (reference)	1.00		1.00		1.00		1.00		1.00		1.00	
1	1.14	(1.10–1.19)	1.08	(1.01–1.15)	1.04	(0.97–1.12)	1.14	(0.92–1.41)	1.25	(1.17–1.33)	1.39	(1.23–1.58)
2	1.24	(1.14–1.36)	1.17	(1.01–1.35)	1.13	(0.97–1.32)	1.92	(1.32–2.77)	1.40	(1.22–1.60)	1.49	(1.14–1.94)
≥ 3	1.57	(1.38–1.80)	1.60	(1.29–1.98)	1.12	(0.86–1.45)	2.32	(1.33–4.03)	1.85	(1.52–2.26)	1.91	(1.28–2.83)
**ACB**												
0 (reference)	1.00		1.00		1.00		1.00		1.00		1.00	
1	1.12	(1.08–1.15)	1.03	(0.98–1.09)	1.18	(1.11–1.25)	1.05	(0.86–1.26)	1.12	(1.06–1.19)	1.16	(1.04–1.30)
2	1.25	(1.19–1.32)	1.10	(1.01–1.20)	1.28	(1.16–1.40)	1.57	(1.21–2.04)	1.32	(1.21–1.43)	1.36	(1.15–1.62)
≥ 3	1.40	(1.32–1.49)	1.21	(1.09–1.34)	1.36	(1.23–1.51)	2.22	(1.69–2.90)	1.61	(1.46–1.77)	1.71	(1.43–2.06)
**ADS**												
0 (reference)	1.00		1.00		1.00		1.00		1.00		1.00	
1	1.25	(1.21–1.30)	1.15	(1.09–1.22)	1.23	(1.16–1.31)	1.39	(1.15–1.68)	1.35	(1.27–1.43)	1.35	(1.20–1.51)
2	1.35	(1.28–1.43)	1.26	(1.16–1.38)	1.27	(1.16–1.39)	1.56	(1.19–2.05)	1.54	(1.42–1.68)	1.45	(1.22–1.72)
≥ 3	1.61	(1.52–1.72)	1.41	(1.27–1.57)	1.49	(1.34–1.67)	2.55	(1.91–3.40)	1.88	(1.70–2.08)	1.91	(1.57–2.32)

*ED* emergency department, *aOR* adjusted odds ratio, *CI* confidence interval, *KABS* Korean Anticholinergic Burden Scale, *ARS* Anticholinergic Risk Scale, *ACB* Anticholinergic Cognitive Burden scale, *ADS* Anticholinergic Drug Scale

^a^Co-morbid conditions, polypharmacy, excessive polypharmacy, exposure to sedative drugs, warfarin, insulin and digoxin, and more than 1 increase compared to previous month’s anticholinergic burden were adjusted for the multivariate regression models

Table 4 shows the top 20 anticholinergic drugs that contributed to the average daily anticholinergic burden score measured by each four scales in the study population. Three of the scales identified the most frequently prescribed medication was ranitidine, followed by dimenhydrinate or chlorpheniramine. The ACB scale, however, found that hydrochlorothiazide contributed the most to the anticholinergic burden (15.7%), which was not found in other scales. Among the anticholinergic drugs indexed solely in the KABS list, octylonium and trimebutine ranked 11th (2.6%) and 13th (2.3%), respectively.

**Table 4:** The top 20 drugs contributing to anticholinergic burden measured by each scale

KABS			ARS			ACB			ADS		
Score	Drug	%	Score	Drug	%	Score	Drug	%	Score	Drug	%
1	ranitidine^a^	11.4	1	ranitidine^a^	29.8	1	hydrochlorothiazide	15.7	2	ranitidine^a^	24.3
3	dimenhydrinate	8.0	3	chlorpheniramine^a^	18.8	1	ranitidine^a^	10.3	3	dimenhydrinate	8.6
3	chlorpheniramine^a^	7.2	2	cimetidine^a^	12.8	3	dimenhydrinate	7.2	3	chlorpheniramine^a^	7.7
2	tramadol	6.7	3	amitriptyline^a^	4.7	3	chlorpheniramine^a^	6.5	2	cimetidine^a^	5.2
3	solifenacin	6.4	2	cetirizine	4.5	3	solifenacin	5.7	1	furosemide	5.0
2	cimetidine^a^	4.9	1	levodopa	4.3	1	furosemide	4.2	1	isosorbide	4.3
1	furosemide	4.7	2	tolterodine^a^	3.9	1	isosorbide	3.6	1	tramadol	3.6
3	propiverine	3.5	3	hydroxyzine^a^	2.6	3	propiverine	3.1	1	nifedipine	3.3
1	escitalopram	3.2	2	amantadine^a^	1.7	1	escitalopram	2.8	1	alprazolam	3.2
1	alprazolam	3.0	1	paroxetine^a^	1.6	1	nifedipine	2.8	3	tolterodine^a^	2.4
3	octylonium^b^	2.6	3	butylscopolamine^a^	1.4	1	alprazolam	2.7	1	diltiazem	2.3
1	levocetirizine	2.5	2	loratadine	1.2	1	atenolol	2.4	1	famotidine	2.0
1	trimebutine^b^	2.3	2	nortriptyline^a^	1.2	1	levocetirizine	2.3	1	nizatidine	1.9
3	tolterodine^a^	2.3	1	mirtazapine	1.2	1	cimetidine^a^	2.2	3	amitriptyline^a^	1.9
3	fesoterodine	2.0	3	imipramine^a^	1.2	3	tolterodine^a^	2.0	1	prednisolone	1.9
3	amitriptyline^a^	1.8	1	quetiapine^a^	1.1	1	doxazosin	1.8	1	methylprednisolone	1.8
1	prednisolone	1.8	2	baclofen	0.9	3	fesoterodine	1.8	1	diazepam	1.8
1	diazepam	1.6	2	loperamide^a^	0.9	3	paroxetine^a^	1.7	1	digoxin	1.6
1	digoxin	1.5	1	trazodone	0.9	3	amitriptyline^a^	1.6	1	dexamethasone	1.3
2	paroxetine^a^	1.2	2	olanzapine^a^	0.8	1	prednisolone	1.6	1	lorazepam	1.3

*KABS* Korean Anticholinergic Burden Scale, *ARS* Anticholinergic Risk Scale, *ACB* Anticholinergic Cognitive Burden, *ADS* Anticholinergic Drug Scale

^a^Drugs common to all four anticholinergic drug scales

^b^Drugs included only in the KABS list

## Discussion

This study revealed that the anticholinergic burden, when measured with the KABS, identified a higher proportion of high anticholinergic burden as well as a stronger dose-response relationship for overall anticholinergic ED visits and individual anticholinergic adverse effect (delirium and urinary retention) than the three popular anticholinergic scales in older Korean adults.

Similar to the findings from other countries, such as New Zealand [23], Australia [16] and Germany [14], this current study confirmed that different scales identified different proportions of patients with a high anticholinergic burden. The ACB identified the largest proportion of exposure to anticholinergics (58.0%), the ARS captured the least (37.6%), and the KABS captured the largest proportion of older adults with a high anticholinergic burden (5.0%). Despite the similar definition of anticholinergic exposure, prevalent anticholinergic drugs were different in the New Zealand study [23]. Gastrointestinal drugs such as ranitidine, cimetidine, and trimebutine ranked high in the list that contributed to the average daily AB scores in Korea, while antidepressant drugs such as nortriptyline, fluoxetine, and paroxetine ranked high in New Zealand. In Korea, first-generation antihistamines such as dimenhydrinate and chlorpheniramine ranked high in contributing to the anticholinergic burden, but in New Zealand, second-generation antihistamines, such as cetirizine and loratadine were presented as the most commonly prescribed drug.

Our study found the associations between anticholinergic burden and ED visits are in line with those from previous studies, but our results of the comparative analyses among scales are different from earlier results. In a comparative study by Hsu et al., the ACB had the highest dose-dependent correlation with all-cause ED visits [15]. In contrast, our study showed that the ACB had the lowest correlation with ED visits due to anticholinergic adverse events. This discrepancy could be explained by the differences in prescribing practices as well as the availability of the various medications in the different countries. Mayer et al. reviewed published studies that evaluated the association of anticholinergic burden measured using different scales with patient reported outcomes, and found the results differed considerably depending on which scale was used [14]. Those findings implicated that the choice of anticholinergic burden assessment tools is an important issue in clinical applications, as the predictive validity for negative clinical outcomes may depend on the tool applied [10, 11, 14, 16, 24, 25].

To the best of our knowledge, this study is the first to show that a country-specific anticholinergic burden scale was superior in identifying patients with a high anticholinergic burden as having a higher association with acute anticholinergic adverse effects to the other scales developed in different countries. In addition, this study supplemented evidence that anticholinergic burden was associated with severe urinary retention and constipation leading to ED visits, whereas most previous studies focused on central nervous system effects, such as cognitive impairment and dementia, or falls [26, 27].

There are several limitations to be considered. First, similar to previous studies, there are inherent limitations of claims data analyses such as over-the-counter medications may not be included, cannot validate whether medications are actually taken, and the inability to take into account laboratory findings such as kidney or liver function [20]. Second, as we selected control using propensity score matching with minimum matching variables, selection bias due to residual confounding inherent in case-control study might affect the study results even though we included unbalanced variables in regression for adjustment. Finally, this study validated the KABS focusing on anticholinergic adverse effects that may occur with short-term use in older adults. Validation of the KABS regarding the prediction of longer-term negative clinical outcomes of anticholinergic burden, such as cognitive impairment and dementia, should be conducted.

## Conclusion

In conclusion, this study demonstrated that the country-specific anticholinergic burden scale, KABS, is superior to previously developed scales for identifying patients with high anticholinergic burden as having high association with ED visits secondary to anticholinergic adverse events in older Korean adults. The KABS might be a practical scale for assessing anticholinergic burden and subsequently deprescribing inappropriate medications to prevent anticholinergic complications.

## Supplementary Information

**Additional file 1.** Diagnostic codes used to identify anticholinergic adverse outcomes and comorbidities.

**Additional file 2.** The comparable list of anticholinergics in four scales.

## Data Availability

The data that support the findings of this study are available from Health Insurance Review and Assessment Service (HIRA) but restrictions apply to the availability of these data, which were used under license for the current study, and so are not publicly available. Data are however available from the authors upon reasonable request and with permission of HIRA.

## Abbreviations

KABS: Korean Anticholinergic Burden Scale
AB: Anticholinergic Burden
ED: Emergency Department
ARS: Anticholinergic Risk Scale
ACB: Anticholinergic Cognitive Burden Scale
ADS: Anticholinergic Drug Scale
HIRA: Health Insurance Review and Assessment Service
APS: Adult Patient Sample
DDD: Defined Daily Dose
WHO: World Health Organization
CCI: Charlson Comorbidity Index
aOR: adjusted Odds Ratio
CI: Confidence Interval
