# Supplementary material for: Comparative associations between anticholinergic burden and emergency department visits for anticholinergic adverse events in older Korean adults: a nested case-control study using national claims data for validation of a novel country-specific scale
Source: BMC Pharmacol Toxicol. 2021 Jan 7;22:2. doi: 10.1186/s40360-020-00467-6 (PMC7792041; doi:10.1186/s40360-020-00467-6)
Supplement: Supplementary file 1 — Additional file 1. Diagnostic codes used to identify anticholinergic adverse outcomes and comorbidities. [file 40360_2020_467_MOESM1_ESM.docx]

Supplementary Table 1. Diagnostic codes used to identify anticholinergic adverse outcomes and comorbidities

| **Anticholinergic adverse outcome** | **International Classification of Diseases (10th version codes)** |
| --- | --- |
| Fall | W00-W19 |
| Fracture | S02, S12, S22, S32, S42, S52, S62, S72, S82,  S92, T02, T08, T10, T12, T14.2 |
| Dizziness | R42 |
| Delirium | F05 |
| Constipation | K59.0 |
| Urinary retention | R33, R39.1 |
| **Comorbidity** | **International Classification of Diseases (10th version codes)** |
| Diabetes Mellitus | E10-14 |
| Chronic obstructive pulmonary disease | J4, J60-67, J68.4, J70.1, J70.3, I27.8, I27.9 |
| Cerebrovascular disease | I60-69 |
| Coronary artery disease | I20-25 |
| Liver failure | K70, K71.1, K71.3, K71.4, K71.5, K71.7, K72,  K73, K74, K76, I85, I86.4, I98.2 |
| Congestive heart failure | I50, I11.0, I13.0, I13.2 |
| Cancer | C% |
| Renal failure | N18, N19, N03, N05.2-N05.7, N25.0, Z49, Z99.2, I12.0, I13.1 |
| Parkinson | G20-22 |
