# Supplementary material for: Comparative associations between anticholinergic burden and emergency department visits for anticholinergic adverse events in older Korean adults: a nested case-control study using national claims data for validation of a novel country-specific scale
Source: BMC Pharmacol Toxicol. 2021 Jan 7;22:2. doi: 10.1186/s40360-020-00467-6 (PMC7792041; doi:10.1186/s40360-020-00467-6)
Supplement: Supplementary file 2 — Additional file 2. The comparable list of anticholinergics in four scales. [file 40360_2020_467_MOESM2_ESM.docx]

Supplementary Table 2. The comparable list of anticholinergics in four scales

| **No.** | **Category** | **Agent** | **KABS** | **ARS** | **ACB** | **ADS** |
| --- | --- | --- | --- | --- | --- | --- |
| 1 | 1 | amantadine | 2 | 2 | 2 | 1 |
| 2 | 1 | amitriptyline | 3 | 3 | 3 | 3 |
| 3 | 1 | atropine | 3 | 3 | 3 | 3 |
| 4 | 1 | chlorpheniramine | 3 | 3 | 3 | 3 |
| 5 | 1 | chlorpromazine | 3 | 3 | 3 | 3 |
| 6 | 1 | cimetidine | 2 | 2 | 1 | 2 |
| 7 | 1 | clozapine | 3 | 2 | 3 | 3 |
| 8 | 1 | cyclobenzaprine | 2 | 2 | 2 | 2 |
| 9 | 1 | cyproheptadine | 2 | 3 | 2 | 2 |
| 10 | 1 | dicycloverine | 3 | 3 | 3 | 3 |
| 11 | 1 | diphenhydramine | 3 | 3 | 3 | 3 |
| 12 | 1 | hydroxyzine | 3 | 3 | 3 | 3 |
| 13 | 1 | imipramine | 3 | 3 | 3 | 3 |
| 14 | 1 | loperamide | 1 | 2 | 1 | 1 |
| 15 | 1 | meclizine | 3 | 3 | 3 | 3 |
| 16 | 1 | nortriptyline | 3 | 2 | 3 | 3 |
| 17 | 1 | olanzapine | 3 | 2 | 3 | 1 |
| 18 | 1 | oxybutynin | 3 | 3 | 3 | 3 |
| 19 | 1 | paroxetine | 2 | 1 | 3 | 1 |
| 20 | 1 | perphenazine | 2 | 3 | 3 | 1 |
| 21 | 1 | quetiapine | 2 | 1 | 3 | 1 |
| 22 | 1 | ranitidine | 1 | 1 | 1 | 2 |
| 23 | 1 | risperidone | 1 | 1 | 1 | 1 |
| 24 | 1 | scopolamine | 3 | 3 | 3 | 3 |
| 25 | 1 | thioridazine | 3 | 3 | 3 | 3 |
| 26 | 1 | tolterodine | 3 | 2 | 3 | 3 |
| 27 | 2 | alprazolam | 1 | 0 | 1 | 1 |
| 28 | 2 | baclofen | 1 | 2 | 1 | 0 |
| 29 | 2 | benztropine | 3 | 3 | 3 | 0 |
| 30 | 2 | brompheniramine | 3 | 0 | 3 | 3 |
| 31 | 2 | carbamazepine | 1 | 0 | 2 | 2 |
| 32 | 2 | carbinoxamine | 3 | 0 | 3 | 3 |
| 33 | 2 | cetirizine | 1 | 2 | 1 | 0 |
| 34 | 2 | clemastine | 3 | 0 | 3 | 3 |
| 35 | 2 | clomipramine | 3 | 0 | 3 | 3 |
| 36 | 2 | clorazepate | 1 | 0 | 1 | 1 |
| 37 | 2 | codeine | 1 | 0 | 1 | 1 |
| 38 | 2 | diazepam | 1 | 0 | 1 | 1 |
| 39 | 2 | digoxin | 1 | 0 | 1 | 1 |
| 40 | 2 | dimenhydrinate | 3 | 0 | 3 | 3 |
| 41 | 2 | doxepin | 3 | 0 | 3 | 3 |
| 42 | 2 | fentanyl | 1 | 0 | 1 | 1 |
| 43 | 2 | flavoxate | 3 | 0 | 3 | 3 |
| 44 | 2 | fluvoxamine | 1 | 0 | 1 | 1 |
| 45 | 2 | furosemide | 1 | 0 | 1 | 1 |
| 46 | 2 | haloperidol | 1 | 1 | 1 | 0 |
| 47 | 2 | hydralazine | 1 | 0 | 1 | 1 |
| 48 | 2 | hydrocortisone | 1 | 0 | 1 | 1 |
| 49 | 2 | loratadine | 1 | 2 | 1 | 0 |
| 50 | 2 | loxapine | 2 | 0 | 2 | 2 |
| 51 | 2 | methocarbamol | 1 | 1 | 0 | 1 |
| 52 | 2 | mirtazapine | 1 | 1 | 1 | 0 |
| 53 | 2 | molindone | 2 | 0 | 2 | 2 |
| 54 | 2 | morphine | 1 | 0 | 1 | 1 |
| 55 | 2 | orphenadrine | 3 | 0 | 3 | 3 |
| 56 | 2 | oxcarbazepine | 2 | 0 | 2 | 2 |
| 57 | 2 | prednisolone | 1 | 0 | 1 | 1 |
| 58 | 2 | prochlorperazine | 0 | 2 | 1 | 1 |
| 59 | 2 | procyclidine | 3 | 0 | 3 | 3 |
| 60 | 2 | promethazine | 0 | 3 | 3 | 3 |
| 61 | 2 | pyrilamine | 3 | 0 | 3 | 3 |
| 62 | 2 | theophylline | 1 | 0 | 1 | 1 |
| 63 | 2 | thiothixene | 1 | 3 | 0 | 1 |
| 64 | 2 | tizanidine | 2 | 3 | 2 | 0 |
| 65 | 2 | trazodone | 1 | 1 | 1 | 0 |
| 66 | 2 | triamterene | 1 | 0 | 1 | 1 |
| 67 | 2 | trifluoperazine | 0 | 3 | 3 | 1 |
| 68 | 2 | trihexyphenidyl | 3 | 0 | 3 | 3 |
| 69 | 3 | alimemazine | 1 | 0 | 1 | 0 |
| 70 | 3 | amisulpride | 1 | 0 | 1 | 0 |
| 71 | 3 | amoxapine | 3 | 0 | 3 | 0 |
| 72 | 3 | aripiprazole | 1 | 0 | 1 | 0 |
| 73 | 3 | belladonna alkaloid | 3 | 0 | 2 | 0 |
| 74 | 3 | biperiden | 3 | 0 | 3 | 0 |
| 75 | 3 | bupropion | 1 | 0 | 1 | 0 |
| 76 | 3 | captopril | 0 | 0 | 1 | 1 |
| 77 | 3 | chlordiazepoxide | 1 | 0 | 0 | 1 |
| 78 | 3 | chlorthalidone | 0 | 0 | 1 | 1 |
| 79 | 3 | cinnarizine | 1 | 0 | 1 | 0 |
| 80 | 3 | citalopram | 1 | 0 | 1 | 0 |
| 81 | 3 | clidinium | 2 | 0 | 1 | 0 |
| 82 | 3 | clonazepam | 1 | 0 | 0 | 1 |
| 83 | 3 | desloratadine | 1 | 0 | 1 | 0 |
| 84 | 3 | desvenlafaxine | 1 | 0 | 1 | 0 |
| 85 | 3 | dexchlorpheniramine | 3 | 0 | 3 | 0 |
| 86 | 3 | dipyridamole | 0 | 0 | 1 | 1 |
| 87 | 3 | disopyramide | 0 | 0 | 1 | 2 |
| 88 | 3 | doxylamine | 3 | 0 | 3 | 0 |
| 89 | 3 | escitalopram | 1 | 0 | 1 | 0 |
| 90 | 3 | estazolam | 1 | 0 | 0 | 1 |
| 91 | 3 | fesoterodine | 3 | 0 | 3 | 0 |
| 92 | 3 | fluoxetine | 1 | 0 | 0 | 1 |
| 93 | 3 | flupentixol | 1 | 0 | 1 | 0 |
| 94 | 3 | flurazepam | 1 | 0 | 0 | 1 |
| 95 | 3 | isosorbide | 0 | 0 | 1 | 1 |
| 96 | 3 | ketotifen | 1 | 0 | 0 | 1 |
| 97 | 3 | levocetirizine | 1 | 0 | 1 | 0 |
| 98 | 3 | levomepromazine | 2 | 0 | 2 | 0 |
| 99 | 3 | lorazepam | 1 | 0 | 0 | 1 |
| 100 | 3 | midazolam | 1 | 0 | 0 | 1 |
| 101 | 3 | nefopam | 2 | 0 | 2 | 0 |
| 102 | 3 | nifedipine | 0 | 0 | 1 | 1 |
| 103 | 3 | oxycodone | 1 | 0 | 0 | 1 |
| 104 | 3 | paliperidone | 1 | 0 | 1 | 0 |
| 105 | 3 | pethidine | 2 | 0 | 2 | 0 |
| 106 | 3 | pimozide | 2 | 0 | 0 | 2 |
| 107 | 3 | propiverine | 3 | 0 | 3 | 0 |
| 108 | 3 | solifenacin | 3 | 0 | 3 | 0 |
| 109 | 3 | sulpiride | 1 | 0 | 1 | 0 |
| 110 | 3 | temazepam | 1 | 0 | 0 | 1 |
| 111 | 3 | tramadol | 2 | 0 | 0 | 1 |
| 112 | 3 | triazolam | 1 | 0 | 0 | 1 |
| 113 | 3 | triprolidine | 2 | 2 | 0 | 0 |
| 114 | 3 | trospium | 3 | 0 | 3 | 0 |
| 115 | 3 | venlafaxine | 1 | 0 | 1 | 0 |
| 116 | 3 | warfarin | 0 | 0 | 1 | 1 |
| 117 | 3 | ziprasidone | 1 | 1 | 0 | 0 |
| 118 | 3 | zuclopenthixol | 2 | 0 | 2 | 0 |
| 119 | 4 | blonanserin* | 1 | 0 | 0 | 0 |
| 120 | 4 | bromperidol* | 1 | 0 | 0 | 0 |
| 121 | 4 | chlorprothixene | 3 | 0 | 0 | 0 |
| 122 | 4 | cimetropium* | 3 | 0 | 0 | 0 |
| 123 | 4 | cloperastine* | 2 | 0 | 0 | 0 |
| 124 | 4 | dexbrompheniramine | 3 | 0 | 0 | 0 |
| 125 | 4 | dextromethorphan | 1 | 0 | 0 | 0 |
| 126 | 4 | difemerine* | 3 | 0 | 0 | 0 |
| 127 | 4 | difenidol* | 2 | 0 | 0 | 0 |
| 128 | 4 | emedastine* | 1 | 0 | 0 | 0 |
| 129 | 4 | flunitrazepam | 1 | 0 | 0 | 0 |
| 130 | 4 | glycopyrrolate | 2 | 0 | 0 | 0 |
| 131 | 4 | guaifenesin | 1 | 0 | 0 | 0 |
| 132 | 4 | homochlorcyclizine* | 3 | 0 | 0 | 0 |
| 133 | 4 | hydrocodone | 1 | 0 | 0 | 0 |
| 134 | 4 | imidafenacin* | 3 | 0 | 0 | 0 |
| 135 | 4 | mebeverine | 1 | 0 | 0 | 0 |
| 136 | 4 | mequitazine* | 3 | 0 | 0 | 0 |
| 137 | 4 | octylonium* | 3 | 0 | 0 | 0 |
| 138 | 4 | oxapium* | 3 | 0 | 0 | 0 |
| 139 | 4 | pheniramine | 3 | 0 | 0 | 0 |
| 140 | 4 | piprinhydrinate* | 3 | 0 | 0 | 0 |
| 141 | 4 | pridinol* | 3 | 0 | 0 | 0 |
| 142 | 4 | scopolia extract* | 3 | 0 | 0 | 0 |
| 143 | 4 | tiemonium* | 3 | 0 | 0 | 0 |
| 144 | 4 | timepidium* | 3 | 0 | 0 | 0 |
| 145 | 4 | tiquizium* | 3 | 0 | 0 | 0 |
| 146 | 4 | trimebutine* | 1 | 0 | 0 | 0 |
| 147 | 4 | valethamate* | 3 | 0 | 0 | 0 |
| 148 | 4 | zotepine* | 2 | 0 | 0 | 0 |
| 149 | 5 | carisoprodol | 0 | 3 | 0 | 0 |
| 150 | 5 | entacapone | 0 | 1 | 0 | 0 |
| 151 | 5 | levodopa | 0 | 1 | 0 | 0 |
| 152 | 5 | metoclopramide | 0 | 1 | 0 | 0 |
| 153 | 5 | pramipexole | 0 | 1 | 0 | 0 |
| 154 | 5 | selegiline | 0 | 1 | 0 | 0 |
| 155 | 6 | alverine | 0 | 0 | 1 | 0 |
| 156 | 6 | atenolol | 0 | 0 | 1 | 0 |
| 157 | 6 | bendroflumethiazide | 0 | 0 | 1 | 0 |
| 158 | 6 | colchicine | 0 | 0 | 1 | 0 |
| 159 | 6 | doxazosin | 0 | 0 | 1 | 0 |
| 160 | 6 | hydrochlorothiazide | 0 | 0 | 1 | 0 |
| 161 | 6 | metoprolol | 0 | 0 | 1 | 0 |
| 162 | 6 | quinidine | 0 | 0 | 1 | 0 |
| 163 | 7 | ampicillin | 0 | 0 | 0 | 1 |
| 164 | 7 | azathioprine | 0 | 0 | 0 | 1 |
| 165 | 7 | bromocriptine | 0 | 0 | 0 | 1 |
| 166 | 7 | cefamandole | 0 | 0 | 0 | 1 |
| 167 | 7 | cefoxitin | 0 | 0 | 0 | 1 |
| 168 | 7 | cephalothin | 0 | 0 | 0 | 1 |
| 169 | 7 | clindamycin | 0 | 0 | 0 | 1 |
| 170 | 7 | cycloserine | 0 | 0 | 0 | 1 |
| 171 | 7 | cyclosporine | 0 | 0 | 0 | 1 |
| 172 | 7 | dexamethasone | 0 | 0 | 0 | 1 |
| 173 | 7 | diltiazem | 0 | 0 | 0 | 1 |
| 174 | 7 | famotidine | 0 | 0 | 0 | 1 |
| 175 | 7 | gentamicin | 0 | 0 | 0 | 1 |
| 176 | 7 | methylprednisolone | 0 | 0 | 0 | 1 |
| 177 | 7 | nizatidine | 0 | 0 | 0 | 1 |
| 178 | 7 | oxazepam | 0 | 0 | 0 | 1 |
| 179 | 7 | pancuronium | 0 | 0 | 0 | 1 |
| 180 | 7 | piperacillin | 0 | 0 | 0 | 1 |
| 181 | 7 | sertraline | 0 | 0 | 0 | 1 |
| 182 | 7 | topiramate | 0 | 0 | 0 | 1 |
| 183 | 7 | triamcinolone | 0 | 0 | 0 | 1 |
| 184 | 7 | valproic acid | 0 | 0 | 0 | 1 |
| 185 | 7 | vancomycin | 0 | 0 | 0 | 1 |

KABS, Korean Anticholinergic Burden Scale; ARS, Anticholinergic Risk Scale; ACB, Anticholinergic Cognitive Burden scale; ADS, Anticholinergic Drug Scale. Category 1: Anticholinergics common to all scales, Category 2: Anticholinergics common to three scales, Category 3: Anticholinergics common to two scales, Category 4: Anticholinergics only in KABS, Category 5: Anticholinergics only in ARS, Category 6: Anticholinergics only in ACB, Category 7: Anticholinergics only in ADS *Marketed in Korea and newly added to KABS after literature review and the Delphi process.
